# *catena*-Poly[[cyclo­hexyl­diphenyl­tin(IV)]-μ-hydroxido-κ^2^ *O*:*O*]

**DOI:** 10.1107/S1600536808011100

**Published:** 2008-04-26

**Authors:** Kong Mun Lo, Seik Weng Ng

**Affiliations:** a Department of Chemistry, University of Malaya, 50603 Kuala Lumpur, Malaysia

## Abstract

The title polymeric mixed-organyl tin hydroxide, [Sn(C_6_H_5_)_2_(C_6_H_11_)(OH)]_*n*_, hass a hydroxide-bridged chain structure; the tin center shows *trans*-C_3_SnO_2_ trigonal bipyramidal coordination. The Sn atom lies on a special position of site symmetry *m*; the symmetry element relates one phenyl ring to the other and also relates one half of the cyclo­hexyl ring to the other half.

## Related literature

For background literature on mixed alk­yl/diaryltin(IV) compounds, see: Koshy *et al.* (2001 ▶). For the synthesis of cyclo­hexyl­diphenyl­tin hydroxide, see: Teo *et al.* (2007 ▶). For the structure of triethyl­tin hydroxide, see: Deacon *et al.* (1993 ▶). For the structure of tribenzyl­tin hydroxide, see: Chen *et al.* (2005 ▶); Reuter (2004 ▶). For the structure of triphenyl­tin hydroxide, see: Fu *et al.* (2003 ▶); Glidewell & Liles (1978 ▶); Glidewell *et al.* (2002 ▶). For the structure of the mixed organyl compound, benzyl­dimethyl­tin hydroxide, see: Wannagat *et al.* (1993 ▶).
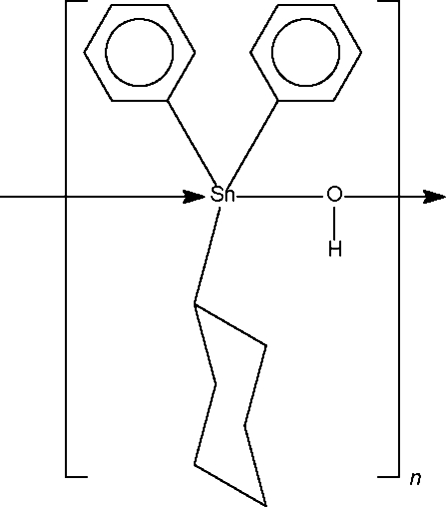


## Experimental

### 

#### Crystal data

[Sn(C_6_H_5_)_2_(C_6_H_11_)(OH)]
*M* *_r_* = 373.05
Orthorhombic, 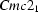

*a* = 18.3830 (2) Å
*b* = 10.2801 (1) Å
*c* = 8.1762 (1) Å
*V* = 1545.13 (3) Å^3^
*Z* = 4
Mo *K*α radiation
μ = 1.65 mm^−1^
*T* = 100 (2) K
0.22 × 0.09 × 0.08 mm

#### Data collection

Bruker SMART APEX diffractometer
Absorption correction: multi-scan (*SADABS*; Sheldrick, 1996 ▶) *T*_min_ = 0.771, *T*_max_ = 0.880
9651 measured reflections
1711 independent reflections
1637 reflections with *I* > 2σ(*I*)
*R*_int_ = 0.024

#### Refinement

*R*[*F* ^2^ > 2σ(*F* ^2^)] = 0.017
*wR*(*F* ^2^) = 0.071
*S* = 1.28
1711 reflections
97 parameters
1 restraint
H-atom parameters constrained
Δρ_max_ = 0.57 e Å^−3^
Δρ_min_ = −0.31 e Å^−3^
Absolute structure: Flack (1983 ▶), 650 Friedel pairs
Flack parameter: 0.02 (4)

### 

Data collection: *APEX2* (Bruker, 2007 ▶); cell refinement: *SAINT* (Bruker, 2007 ▶); data reduction: *SAINT*; program(s) used to solve structure: *SHELXS97* (Sheldrick, 2008 ▶); program(s) used to refine structure: *SHELXL97* (Sheldrick, 2008 ▶); molecular graphics: *X-SEED* (Barbour, 2001 ▶); software used to prepare material for publication: *publCIF* (Westrip, 2008 ▶).

## Supplementary Material

Crystal structure: contains datablocks global, I. DOI: 10.1107/S1600536808011100/tk2257sup1.cif

Structure factors: contains datablocks I. DOI: 10.1107/S1600536808011100/tk2257Isup2.hkl

Additional supplementary materials:  crystallographic information; 3D view; checkCIF report

## Figures and Tables

**Table:** 

Sn1—O1	2.201 (4)
Sn1—C1	2.159 (4)
Sn1—C5	2.139 (3)

**Table:** 

C1—Sn1—C5	118.4 (1)
C1—Sn1—O1	94.1 (2)
C1—Sn1—O1^i^	89.8 (2)
C5—Sn1—C5^ii^	122.9 (2)
C5—Sn1—O1	90.7 (1)
C5—Sn1—O1^i^	87.5 (1)
O1—Sn1—O1^i^	176.1 (1)
Sn1—O1—Sn1^iii^	133.7 (2)

Symmetry codes: (i) 
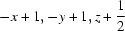
; (ii) 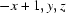; (iii) 
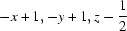
.

**Fig. 1.**
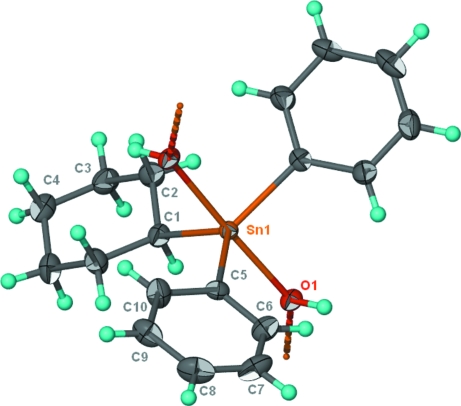
70% Probability thermal ellipsoid plot of the asymmetric unit in Sn(C6H11)(C6H5)2(OH) (I) extended to show the trans-C3SnO2 trigonal bipyramidal coordination geometry. Hydrogen atoms are drawn as spheres of arbitrary radius; symmetry-related atoms are not labeled.

**Fig. 2.**
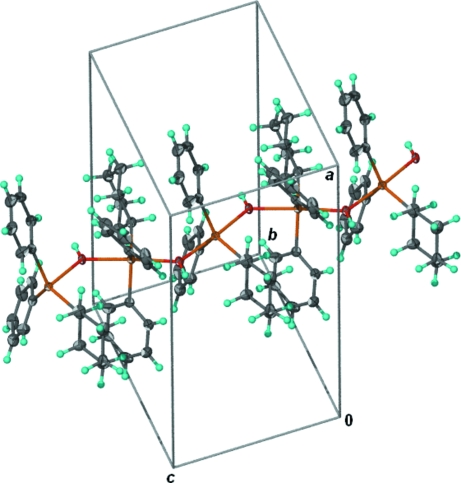
Hydroxo-bridged chain motif in (I).

## supplementary crystallographic information

### Comment

Mixed alkyl/diaryltin compounds possess much more useful activity against plant pathogens than the symmetrical triorganotin homologs, particularly if one of the alkyl substituent is a cyclic unit (Koshy *et al.*, 2001). The title compound (I) is the starting reactant for the synthesis of mixed organotin carboxylates.

The compound adopts a zigzag chain motif that propagates along the *c*-axis of the orthorhombic unit cell; the tin center shows *trans*-C_3_SnO_2_ trigonal bipyramidal coordination (Figs 1 and 2 & Table 1).

### Experimental

The compound was synthesized as described previously (Teo *et al.*, 2007). Crystals were obtained by recrystallization from ethanol.

### Refinement

Carbon-bound H-atoms were placed in calculated positions (C—H 0.95 to 0.99 Å) and were included in the refinement in the riding model approximation, with *U*(H) set to 1.2*U*_eq_(C). The hydroxo H atom (O–H 0.84 Å) was similarly treated.

### Crystal data

**Table:**

[Sn(C_6_H_5_)_2_(C_6_H_11_)(OH)]	*F*_000_ = 752
*M**_r_* = 373.05	*D*_x_ = 1.604 Mg m^−^^3^
Orthorhombic, *C**m**c*2_1_	Mo *K*α radiation λ = 0.71073 Å
Hall symbol: C 2c -2	Cell parameters from 8850 reflections
*a* = 18.3830 (2) Å	θ = 2.2–28.3º
*b* = 10.2801 (1) Å	µ = 1.65 mm^−^^1^
*c* = 8.1762 (1) Å	*T* = 100 (2) K
*V* = 1545.13 (3) Å^3^	Prism, colorless
*Z* = 4	0.22 × 0.09 × 0.08 mm

### Data collection

**Table:**

Bruker SMART APEXII diffractometer	1711 independent reflections
Radiation source: fine-focus sealed tube	1637 reflections with *I* > 2σ(*I*)
Monochromator: graphite	*R*_int_ = 0.024
*T* = 100(2) K	θ_max_ = 27.5º
ω scans	θ_min_ = 2.2º
Absorption correction: multi-scan(SADABS; Sheldrick, 1996)	*h* = −23→23
*T*_min_ = 0.771, *T*_max_ = 0.880	*k* = −13→13
9651 measured reflections	*l* = −9→10

### Refinement

**Table:**

Refinement on *F*^2^	Hydrogen site location: inferred from neighbouring sites
Least-squares matrix: full	H-atom parameters constrained
*R*[*F*^2^ > 2σ(*F*^2^)] = 0.017	*w* = 1/[σ^2^(*F*_o_^2^) + (0.0421*P*)^2^ + 0.0692*P*] where *P* = (*F*_o_^2^ + 2*F*_c_^2^)/3
*wR*(*F*^2^) = 0.071	(Δ/σ)_max_ = 0.001
*S* = 1.28	Δρ_max_ = 0.57 e Å^−^^3^
1711 reflections	Δρ_min_ = −0.31 e Å^−^^3^
97 parameters	Extinction correction: none
1 restraint	Absolute structure: Flack (1983), 650 Friedel pairs
Primary atom site location: structure-invariant direct methods	Flack parameter: 0.02 (4)
Secondary atom site location: difference Fourier map	

### Fractional atomic coordinates and isotropic or equivalent isotropic displacement parameters (Å^2^)

**Table:**

	*x*	*y*	*z*	*U*_iso_*/*U*_eq_	
Sn1	0.5000	0.494121 (18)	0.50000 (18)	0.01298 (10)	
O1	0.5000	0.5850 (3)	0.2563 (4)	0.0165 (6)	
H1O	0.5000	0.6666	0.2598	0.025*	
C1	0.5000	0.2980 (4)	0.4053 (6)	0.0189 (9)	
H1	0.5000	0.3071	0.2836	0.023*	
C2	0.43184 (16)	0.2226 (3)	0.4465 (5)	0.0234 (7)	
H2A	0.3888	0.2721	0.4084	0.028*	
H2B	0.4281	0.2132	0.5667	0.028*	
C3	0.4313 (2)	0.0883 (3)	0.3681 (5)	0.0260 (8)	
H3A	0.3885	0.0392	0.4078	0.031*	
H3B	0.4268	0.0977	0.2480	0.031*	
C4	0.5000	0.0125 (4)	0.4074 (8)	0.0248 (13)	
H4A	0.5000	−0.0102	0.5251	0.030*	
H4B	0.5000	−0.0696	0.3442	0.030*	
C5	0.60222 (16)	0.5831 (3)	0.5558 (4)	0.0170 (6)	
C6	0.62261 (15)	0.7060 (2)	0.5004 (5)	0.0227 (6)	
H6	0.5897	0.7548	0.4352	0.027*	
C7	0.69028 (19)	0.7587 (3)	0.5387 (4)	0.0296 (8)	
H7	0.7023	0.8441	0.5036	0.036*	
C8	0.74004 (18)	0.6867 (4)	0.6280 (5)	0.0308 (8)	
H8	0.7867	0.7216	0.6514	0.037*	
C9	0.72163 (17)	0.5644 (4)	0.6826 (5)	0.0256 (7)	
H9	0.7553	0.5149	0.7449	0.031*	
C10	0.6529 (3)	0.5133 (3)	0.6458 (7)	0.0247 (9)	
H10	0.6407	0.4287	0.6836	0.030*	

### Atomic displacement parameters (Å^2^)

**Table:**

	*U*^11^	*U*^22^	*U*^33^	*U*^12^	*U*^13^	*U*^23^
Sn1	0.01281 (14)	0.01401 (14)	0.01211 (16)	0.000	0.000	0.0003 (2)
O1	0.0227 (14)	0.0168 (15)	0.0101 (15)	0.000	0.000	−0.0008 (11)
C1	0.022 (2)	0.0167 (19)	0.018 (2)	0.000	0.000	0.0005 (17)
C2	0.0200 (15)	0.0215 (14)	0.0288 (19)	−0.0023 (11)	0.0003 (13)	−0.0006 (12)
C3	0.0306 (19)	0.0193 (15)	0.028 (2)	−0.0044 (13)	0.0033 (14)	0.0001 (14)
C4	0.038 (4)	0.019 (2)	0.017 (3)	0.000	0.000	−0.0032 (17)
C5	0.0168 (13)	0.0194 (13)	0.0147 (15)	−0.0006 (12)	0.0029 (11)	−0.0018 (11)
C6	0.0268 (14)	0.0242 (12)	0.0171 (16)	−0.0042 (10)	−0.0025 (17)	0.001 (2)
C7	0.0366 (18)	0.0302 (16)	0.022 (2)	−0.0150 (14)	0.0004 (14)	0.0036 (13)
C8	0.0213 (16)	0.043 (2)	0.028 (2)	−0.0107 (14)	−0.0009 (15)	−0.0088 (16)
C9	0.0174 (15)	0.0343 (19)	0.0250 (18)	0.0007 (13)	−0.0047 (13)	−0.0072 (15)
C10	0.023 (2)	0.0195 (17)	0.032 (3)	−0.0007 (11)	−0.0051 (18)	−0.0014 (14)

### Geometric parameters (Å, °)

**Table:**

Sn1—O1	2.201 (4)	C8—C9	1.377 (5)
Sn1—C1	2.159 (4)	C9—C10	1.400 (5)
Sn1—C5	2.139 (3)	O1—H1O	0.8400
Sn1—C5^i^	2.139 (3)	C1—H1	1.0000
Sn1—O1^ii^	2.248 (4)	C2—H2A	0.9900
O1—Sn1^iii^	2.248 (4)	C2—H2B	0.9900
C1—C2	1.511 (4)	C3—H3A	0.9900
C1—C2^i^	1.511 (4)	C3—H3B	0.9900
C2—C3	1.522 (4)	C4—H4A	0.9900
C3—C4	1.518 (4)	C4—H4B	0.9900
C4—C3^i^	1.518 (4)	C6—H6	0.9500
C5—C10	1.388 (6)	C7—H7	0.9500
C5—C6	1.394 (4)	C8—H8	0.9500
C6—C7	1.393 (4)	C9—H9	0.9500
C7—C8	1.385 (5)	C10—H10	0.9500
			
C1—Sn1—C5	118.4 (1)	C2—C1—H1	105.6
C1—Sn1—O1	94.1 (2)	Sn1—C1—H1	105.6
C1—Sn1—O1^ii^	89.8 (2)	C1—C2—H2A	109.2
C5—Sn1—C5^i^	122.9 (2)	C3—C2—H2A	109.2
C5—Sn1—O1	90.7 (1)	C1—C2—H2B	109.2
C5—Sn1—O1^ii^	87.5 (1)	C3—C2—H2B	109.2
C5^i^—Sn1—C1	118.4 (1)	H2A—C2—H2B	107.9
C5^i^—Sn1—O1	90.7 (1)	C4—C3—H3A	109.3
C5^i^—Sn1—O1^ii^	87.5 (1)	C2—C3—H3A	109.3
O1—Sn1—O1^ii^	176.1 (1)	C4—C3—H3B	109.3
Sn1—O1—Sn1^iii^	133.7 (2)	C2—C3—H3B	109.3
C2—C1—C2^i^	112.0 (3)	H3A—C3—H3B	107.9
C2—C1—Sn1	113.5 (2)	C3^i^—C4—H4A	109.1
C2^i^—C1—Sn1	113.5 (2)	C3—C4—H4A	109.1
C1—C2—C3	112.1 (3)	C3^i^—C4—H4B	109.1
C4—C3—C2	111.8 (3)	C3—C4—H4B	109.1
C3^i^—C4—C3	112.5 (4)	H4A—C4—H4B	107.8
C10—C5—C6	117.4 (3)	C7—C6—H6	119.3
C10—C5—Sn1	118.8 (2)	C5—C6—H6	119.3
C6—C5—Sn1	123.7 (2)	C8—C7—H7	120.0
C7—C6—C5	121.3 (3)	C6—C7—H7	120.0
C8—C7—C6	120.0 (3)	C9—C8—H8	120.1
C9—C8—C7	119.8 (3)	C7—C8—H8	120.1
C8—C9—C10	119.6 (4)	C8—C9—H9	120.2
C5—C10—C9	121.7 (3)	C10—C9—H9	120.2
Sn1—O1—H1O	113.2	C5—C10—H10	119.1
Sn1^iii^—O1—H1O	113.2	C9—C10—H10	119.1
			
C5—Sn1—O1—Sn1^iii^	118.53 (8)	C1—Sn1—C5—C10	−47.4 (4)
C5^i^—Sn1—O1—Sn1^iii^	−118.53 (8)	O1—Sn1—C5—C10	−142.4 (3)
C1—Sn1—O1—Sn1^iii^	0.0	O1^ii^—Sn1—C5—C10	41.0 (3)
C5—Sn1—C1—C2	151.7 (2)	C5^i^—Sn1—C5—C6	−56.7 (4)
C5^i^—Sn1—C1—C2	−22.3 (3)	C1—Sn1—C5—C6	129.6 (3)
O1—Sn1—C1—C2	−115.3 (3)	O1—Sn1—C5—C6	34.5 (3)
O1^ii^—Sn1—C1—C2	64.7 (3)	O1^ii^—Sn1—C5—C6	−142.0 (3)
C5—Sn1—C1—C2^i^	22.3 (3)	C10—C5—C6—C7	−2.2 (5)
C5^i^—Sn1—C1—C2^i^	−151.7 (2)	Sn1—C5—C6—C7	−179.2 (3)
O1—Sn1—C1—C2^i^	115.3 (3)	C5—C6—C7—C8	2.6 (5)
O1^ii^—Sn1—C1—C2^i^	−64.7 (3)	C6—C7—C8—C9	−1.8 (6)
C2^i^—C1—C2—C3	−53.8 (5)	C7—C8—C9—C10	0.7 (6)
Sn1—C1—C2—C3	176.0 (3)	C6—C5—C10—C9	1.0 (6)
C1—C2—C3—C4	52.9 (5)	Sn1—C5—C10—C9	178.2 (3)
C2—C3—C4—C3^i^	−52.3 (6)	C8—C9—C10—C5	−0.3 (7)
C5^i^—Sn1—C5—C10	126.3 (3)		

Symmetry codes: (i) −*x*+1, *y*, *z*; (ii) −*x*+1, −*y*+1, *z*+1/2; (iii) −*x*+1, −*y*+1, *z*−1/2.
